# Clinical characteristics and prognosis of acute stroke in pregnancy and puerperium (ASPP) patients and their offspring: a retrospective, observational, nationwide, multicenter study protocol

**DOI:** 10.1186/s41016-025-00396-5

**Published:** 2025-06-04

**Authors:** Zhongji Zhang, Zihan Yin, Tong Liu, Xiaolin Zhang, Qihang Zhang, Junlin Lu, Long Mu, Yang Dong, Juning Liu, Yi Xiao, Yanming Chen, Chenyang Song, Zengguang Wang, Yuheng Liu, Wei Ding, Li Zhang, Huaizhang Shi, Jingtao Qi, Bin Tang, Fei Wang, Pin Guo, Yongjun Tang, Mingsheng Yu, Wenjian Zheng, Qinglong He, Jian Yu, Aihua Zhu, Dazhao Fang, Gang Li, Yu Bai, Yushuang Zhang, Jiaxi Li, Yuli Wang, Faliang Gao, Yonggang Ma, Yifan Liu, Li Ma, Bao Yang, Yahui Zhao, Xun Ye, Qian Zhang, Yan Zhang, Xingju Liu, Jizong Zhao, on behalf of the ASPP Study Group

**Affiliations:** 1 Department of Neurosurgery, Beijing Tiantan Hospital, Capital Medical University, Beijing, 100070 China; 2 China National Clinical Research Center for Neurological Diseases, Beijing, China; 3 Department of Child and Adolescent Health Care, Children’s Hospital of Soochow University, Suzhou, Jiangsu China; 4 Department of Neurosurgery, West China Hospital, Sichuan University, Chengdu, Sichuan China; 5 Department of Neurosurgery, The First Affiliated Hospital of Shandong First Medical University and Shandong Provincial Qianfoshan Hospital, Jinan, Shandong China; 6 Department of Neurosurgery, The First Affiliated Hospital of Zhengzhou University, Zhengzhou, Henan China; 7 Department of Women’s Healthcare, The Affiliated Suzhou Hospital of Nanjing Medical University, Suzhou Municipal Hospital, Suzhou, Jiangsu China; 8 Department of Neurosurgery, The Second Affiliated Hospital of Soochow University, Suzhou, Jiangsu China; 9 Department of Neurosurgery, Tianjin Medical University General Hospital, Tianjin, 300052 China; 10 Department of Neurosurgery, Baotou Central Hospital, Baotou, Inner Mongolia China; 11 Department of Neurosurgery, First Affiliated Hospital Harbin Medical University, Harbin, Heilongjiang, China; 12 Department of Neurosurgery, The First Affiliated Hospital, Jiangxi Medical College, Nanchang University, Nanchang, Jiangxi China; 13 Department of Neurosurgery, The Affiliated Hospital of Qingdao University, Qingdao, Shandong China; 14 Department of Pediatric Respiratory, Xiangya Hospital, Central South University, Changsha, Hunan China; 15 Department of Neurosurgery, Tianjin Huanhu Hospital, Tianjin, 300350 China; 16 Department of Neurosurgery, Shenzhen Second People’s Hospital, The First Affiliated Hospital of Shenzhen University Health Science Center, Shenzhen, Guangdong 518035 China; 17 Department of Neurosurgery, The Second Affiliated Hospital of Hainan Medical University, Haikou, Hainan China; 18 Department of Neurosurgery, The First Affiliated Hospital, University of Science and Technology of China, Hefei, Anhui China; 19 Department of Neurosurgery, Affiliated Hospital of Jiangnan University, Wuxi, Jiangsu China; 20 Department of Neurosurgery, The Affiliated Huaian No.1 People’s Hospital of Nanjing Medical University, Huaian, Jiangsu China; 21 Department of Neurosurgery, Cangzhou Central Hospital, Hebei Medical University, Cangzhou, Hebei China; 22 Department of Neurosurgery, Sanbo Brain Hospital, Capital Medical University, Beijing, China; 23 Department of Pediatric, Guangyuan Central Hospital, Guangyuan, Sichuan China; 24 Department of Neurosurgery, The First Affiliated Hospital of Xi’an Jiaotong University, Xi’an, Shaanxi China; 25 Department of Neurosurgery, Xinxiang Central Hospital, The Fourth Clinical College of Xinxiang Medical University, Xinxiang, Henan 453000 China; 26 Department of Neurosurgery, Zhejiang Provincial People’s Hospital, Hangzhou Medical College, Hangzhou, Zhejiang China; 27 Department of Neurointerventional Surgery, Binzhou Medical University Hospital, Binzhou, Shandong 256603 China; 28 Shanxi Bethune Hospital, Shanxi Academy of Medical Sciences, Tongji Shanxi Hospital, Third Hospital of Shanxi Medical University, Taiyuan, Shanxi 030032 China; 29 Department of Neurological Surgery, University of Pittsburgh Medical Center, Pittsburgh, PA 15213 USA

**Keywords:** Acute stroke, Pregnancy, Puerperium, Clinical characteristics, Prognosis, Offspring

## Abstract

**Background:**

Previous studies on the risk factors and prognosis of acute stroke in pregnancy and puerperium (ASPP) mainly used European and American national healthcare databases, lacking detailed patient-level data and precise event timing.

**Aim:**

(1) To identify the risk factors and prognostic factors for ASPP, (2) to assess the risk of recurrent stroke, particularly during subsequent pregnancies, and (3) to evaluate offspring prognosis.

**Design:**

This study is a retrospective, observational, nationwide, multicenter research project planned to include 400 ASPP patients from 36 centers across 22 provinces in China, from 2015 to 2024. ASPP is defined as acute ischemic or hemorrhagic stroke during pregnancy or within 6 weeks postpartum, confirmed by neuroimaging. Two matched groups will be included for comparison: 400 pregnant/puerperal participants without a stroke history and 400 nonpregnant/puerperal participants with a recent stroke, matched by age and/or stroke etiology.

**Methods:**

All participants will be followed up through telephone interviews. The initial follow-up is scheduled to take place between December 2024 and February 2025. The follow-up phase will consist of three rounds, each lasting 3 months and conducted every 3 years. Primary outcomes include unfavorable functional outcomes (*mRS* > 2 or EQ-5D index score < 0.7) at follow-up for Aim 1, recurrent strokes (neuroimaging-confirmed) for Aim 2, and neonatal asphyxia (Apgar scoring) and future development (ASQ-3) of offspring for Aim 3.

**Discussion:**

The ASPP study is the first nationwide multicenter study to systematically evaluate the risk factors, prognosis, and risk of recurrent stroke in ASPP patients, particularly during subsequent pregnancies. This research may offer new insights into the long-term impacts of pregnancy-related stroke.

**Trial registration:**

ClinicalTrials.gov (NCT06527807).

## Background

Acute stroke in pregnancy and puerperium (ASPP) is a rare but serious complication associated with high morbidity and mortality in women of childbearing age. The incidence of pregnancy-associated stroke is estimated at 30 per 100,000 pregnancies and appears to be rising, with an inhospital mortality rate of 4.2% for pregnant women experiencing acute stroke [1–3]. Compared to their nonpregnant counterparts, pregnant women with acute stroke face a higher risk of deterioration and more complex management needs [4]. Regarding the long-term risk of ASPP, women with pregnancy-associated stroke have lower risks of recurrent ischemic stroke and mortality compared to those with strokes unrelated to pregnancy. Furthermore, the risks of recurrent intracerebral hemorrhage and cerebral venous thrombosis do not differ significantly, and recurrent stroke during subsequent pregnancies remains rare [5].

Previous studies have predominantly utilized large-scale national healthcare data and the International Classification of Diseases (Tenth Revision, Clinical Modification) (ICD-10-CM) to examine the incidence rate and risk factors for ASPP [6–8]. Although these population-based studies benefit from larger sample sizes, they often lack detailed data, which hinders a comprehensive exploration of risk factors and the identification of prognostic predictors and range of variables collected, such as laboratory results, neuroimaging characteristics, and management strategies. This limitation hampers the comprehensive exploration of risk factors and the identification of prognostic predictors. As women with ASPP are generally young and likely to achieve good recovery, no long-term follow-up studies have been conducted to assess their long-term functional outcomes. Additionally, it remains to be explored whether ASPP is solely a short-term risk factor that does not affect a woman’s risk of stroke in future pregnancies or throughout her life.

When pregnant women experience acute strokes, the systemic circulatory system may be severely affected, causing significant fluctuations in blood pressure that impact fetal blood flow [9]. These dramatic changes in fetal blood flow can lead to adverse intrauterine effects, such as fetal distress and neonatal asphyxia, potentially resulting in unfavorable outcomes for the offspring, thereby affecting the prognosis of the offspring [10]. However, no study has explored the impact of acute stroke in pregnancy and puerperium on the offspring and their future development. Besides, AMI (acute myocardial infarction) in pregnancy and ASPP may share a common pathophysiological mechanism, where their onset can be predisposed by physiological changes related to a hypercoagulable state, vascular endothelial remodeling, and alterations in insulin and lipid metabolism [11]. Only a few studies have explored the maternal and fetal outcomes of patients with AMI during pregnancy, and no study has assessed the future development of their offspring, mirroring the current research status of ASPP [12]. Therefore, it is important to understand the maternal and neonatal outcomes of these patients.

Establishing a cohort based on patients with ASPP is essential for addressing the aforementioned issues. Due to the rarity of ASPP, obtaining a sufficient sample size necessitates a nationwide, multicenter study. In 2020, the European Stroke Organisation proposed an international, multicenter, prospective cohort study to comprehensively explore ASPP patients [13]. However, no related study results have been reported to date. Moreover, the study protocol did not include the future development of the offspring of ASPP patients. The objectives of the ASPP study are as follows: (1) to explore the risk factors of ASPP and predictors of its prognosis; (2) to evaluate the risk of recurrent strokes, particularly during subsequent pregnancies, in ASPP patients; and (3) to assess the prognosis of offspring of ASPP patients.

## Methods

### Design

This is a retrospective, observational, nationwide, multicenter study divided into three parts. The first part is a case-control study aimed at identifying the risk factors of ASPP and its prognostic predictors. The second part is a cohort study to evaluate the impact of ASPP on the risk of recurrent stroke. The third part is a cohort study to assess the impact of ASPP on the prognosis of their offspring.

### Participating centers and patient population

**Fig. 1:**
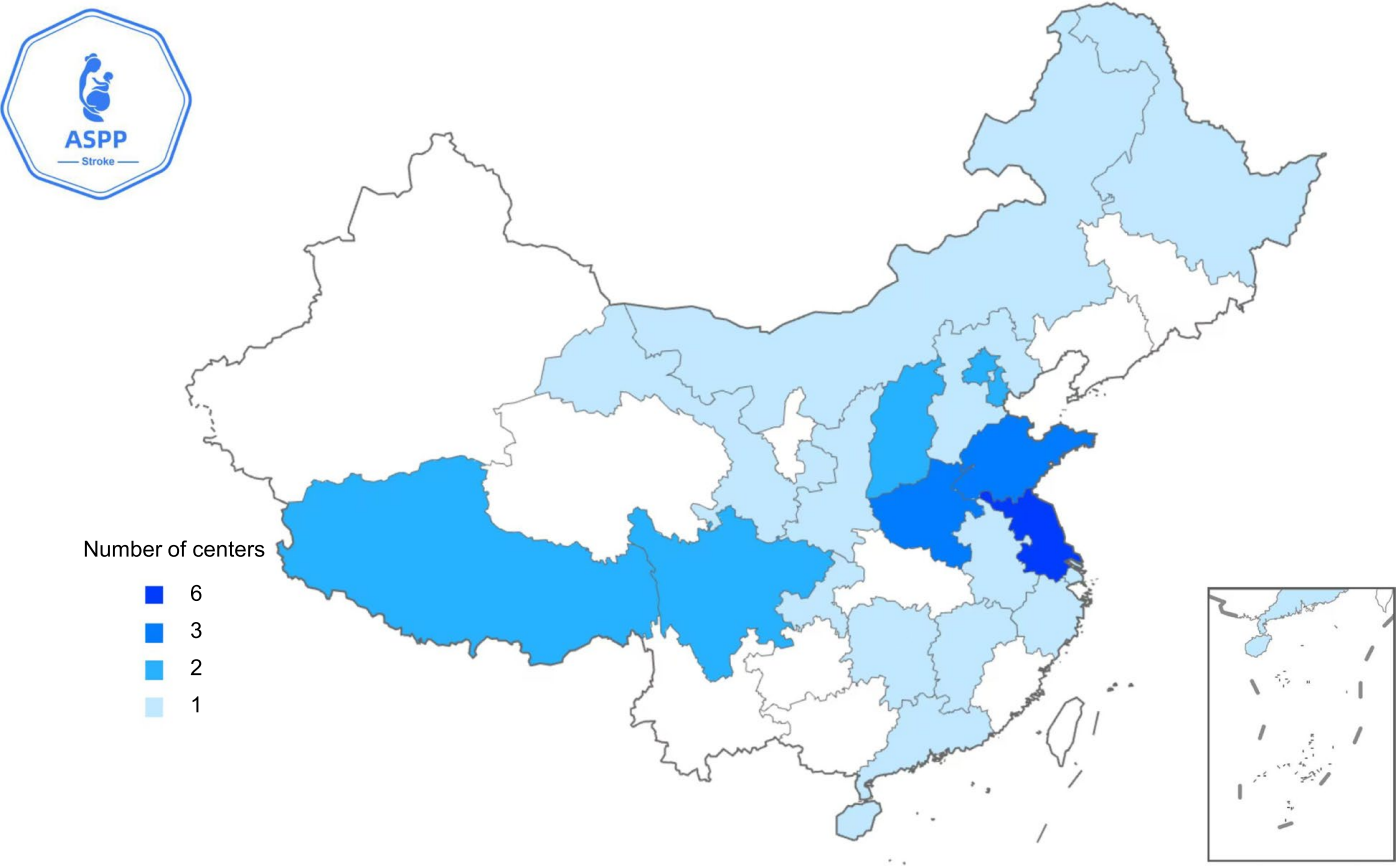
The geographical distribution of centers participating in the ASPP study. The ASPP study aims to include 36 territorial or regional centers across 22 provinces in China

This study is based on the method of stratified selection of centers according to the “China Maternal and Child Health Development Report (2019).” This study plans to include 36 regional or subregional centers in 22 provinces of China that meet 3 criteria to ensure sufficient geographical representativeness, making it a national-level study. The 3 criteria are the incidence rate of ASPP (> 5 cases per 100,000 pregnancies), the minimum annual case volume (≥ 15 ASPP cases), and obtaining standardized stroke care certification. The distribution of the final cohort of 36 tertiary hospitals is strategic and can reflect geographical and socio-economic diversity. Sample allocation uses adaptive quota sampling and is adjusted quarterly. The enrollment targets of each center are dynamically redistributed according to the real-time recruitment rate to maintain balance among the centers. All the above centers were selected based on voluntary participation. The geographical distribution of centers participating in the ASPP study is illustrated in Fig. 1.

**Fig. 2:**
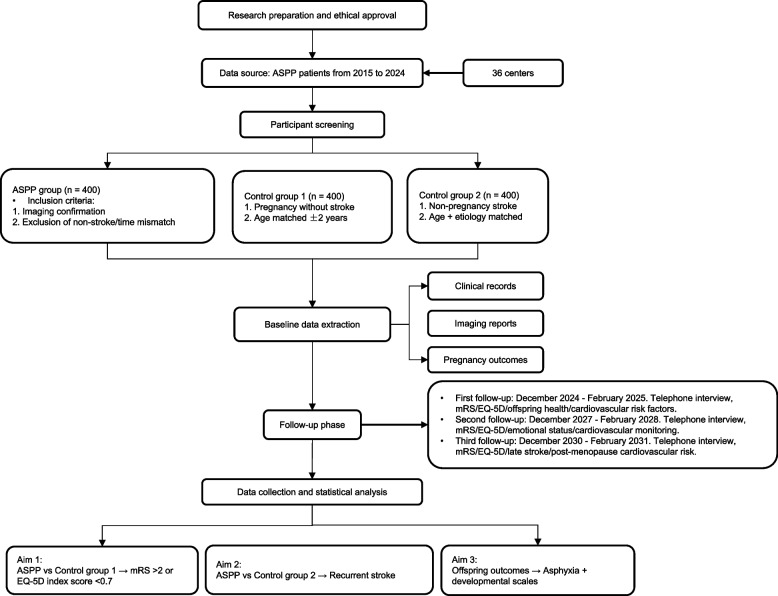
Study design flowchart of the ASPP cohort

ASPP patients and non-ASPP participants from January 2015 to November 2024 will be included (Fig. 2). Table 1 lists the inclusion criteria and exclusion criteria. ASPP patients are defined as those who experience acute stroke events during pregnancy or within 6 weeks postpartum, confirmed by neuroimaging evidence of cerebral infarction or hemorrhage corresponding to their symptoms.

**Table 1:** Inclusion and exclusion criteria

**Inclusion criteria** (1) Aged ≥ 18 years (2) Who are pregnant or within 6 weeks postpartum (3) Patients, their legally acceptable representatives, or relatives who agree to collect personal information for this study and sign an online written informed consent **Exclusion criteria** (1) Missing critical baseline demographic, clinical, or neuroimaging data The key data includes the following: • Age • Body mass index (BMI) • Preexisting conditions (such as a history of hypertension, diabetes, and other chronic diseases) • History of previous strokes • Neuroimaging results (such as CT or MRI findings) (2) Whose offspring have congenital malformations or inborn errors of metabolism (3) With complications affecting cerebral blood flow, such as amniotic fluid embolism during pregnancy (4) The patients’ family members (excluding parents and siblings) have a history of severe mental illness, such as intellectual disability and schizophrenia. In addition, autism spectrum disorder (ASD) is considered a neurodevelopmental disorder, distinct from the traditional definition of mental illness; therefore, it is not included in the exclusion criteria for a family history of severe mental illness (5) Who undergo AMI during pregnancy (6) Who experience non-spontaneous stroke events due to other causes, such as cerebral infarction or hemorrhage caused by surgery	

Grouping of non-ASPP participants is as follows:

1. Non-ASPP participants include two matched control groups: The first group consists of participants in the pregnancy or postpartum period without a history of stroke (pregnancy/postpartum non-stroke group); the second group consists of nonpregnant or non-postpartum participants who have recently experienced a stroke (nonpregnant stroke group).
2. Matching criteria
  For the pregnancy/postpartum non-stroke group, controls will be matched to ASPP cases in a 1:1 ratio based on age (±2 years) and hypertension history, with the latter stratified into preexisting hypertension, pregnancy-induced hypertension, or no hypertension.
  For the nonpregnant stroke group, a 1:1 matching strategy will be applied using age (±2 years), stroke etiology (classified by TOAST criteria), and preexisting hypertension status.
3. Classification of pregnancy-related etiologies is as follows:
  Postpartum vascular disease
  Pregnancy-related hypercoagulable states
  Pregnancy-induced hypertension
  Preeclampsia
  Amniotic fluid embolism
4. Matching of nonpregnant/postpartum patients
  Age matching (±2 years)
  Matching based on stroke etiology, such as cardiogenic stroke, atherosclerotic stroke, small vessel disease stroke, and other identifiable causes (e.g., intracerebral hemorrhage)
  Health status and comorbidities, including common comorbidities such as cardiovascular disease, hypertension, and diabetes
  Lifestyle factors, such as smoking and alcohol consumption history
5. Discussion of the impact of pregnancy-related etiologies
  Etiological characteristics, such as thrombosis resulting from pregnancy-related hypercoagulable states, hemodynamic changes caused by postpartum vascular disease, and hypertensive lesions associated with preeclampsia
  Physiological changes: Physiological changes during pregnancy, such as increased blood volume, blood pressure fluctuations, and alterations in coagulation mechanisms
  Psychological and social factors: Assessing the unique challenges faced by pregnant women in coping with stress, anxiety, and other mental health issues, as well as their potential impact on stroke occurrence
  Impact assessment: When comparing ASPP patients with nonpregnant stroke patients, the influence of pregnancy-related etiologies on stroke recurrence rates and long-term prognosis will be clearly delineated.

### Data collection and management

Baseline data collection will be conducted through the electronic medical record (EMR) system of the participating centers. If certain variables are not recorded in the EMR, they will be collected retrospectively through telephone interviews. Table 2 presents the variables to be collected at baseline. A specialized secure database (CRF, case report form) will be established to facilitate the collection of standardized data from different centers. All data will be anonymized to ensure privacy, and each patient included in this study will be identified by an identity number. General variables (such as demographics, medical history, and medication history) and pregnancy- or puerperium-associated variables (including data on delivery and offspring-related variables) will also be collected for non-ASPP participants.

**Table 2:** Baseline variables

• **Demographics**
▪ Age
▪ Ethnic: Han or other
• **General clinical variables**
▪ Date of admission and discharge
▪ BMI (calculated based on prepregnancy weight and height data)
▪ Previous or current smoking
▪ Previous or current drinking
▪ Previous or current drug abuse
▪ NIHSS and/or mRS at admission and discharge
▪ Blood routine index: RBC, WBC, PLT, etc.
▪ Blood biochemical index: TC, HDL-C, etc.
▪ Coagulation function index: TT, PT, FIB, APTT, etc.
▪ Other blood index: lupus anticoagulant, anticardiolipin antibodies, etc.
• **Medical history**
▪ Hypertension (if with record maximal SBP and DBP)
▪ Diabetes
▪ Migraine
▪ Polycystic kidney disease
▪ Cerebral vascular disease (if with record etiology)
▪ Myocardial infarction or angina
▪ Congestive heart failure
▪ Known atrial fibrillation or flutter
▪ Valvular heart disease
▪ Hypercholesterolemia
▪ Pulmonary embolism
**• Medication history**
▪ Antihypertensive (if with record the duration of antihypertensive medication use, including pre-pregnancy and during pregnancy, and the types of medications, such as methyldopa, labetalol, amlodipine)
▪ Antiplatelet
▪ Oral contraceptive
• **Stroke evaluation**
▪ Type: Ischemic (thrombotic, embolic) or hemorrhagic (intracerebral, subarachnoid)
▪ Etiology
(1) Ischemic stroke (carotid stenosis, intracranial atherosclerotic stenosis, cardiogenic embolic stroke, arterial dissection, pregnancy-related hypercoagulable state, moyamoya disease)
(2) Hemorrhagic stroke (AVM, IA, pregnancy-related hypercoagulable state)
(3) Other stroke types: Cerebral venous thrombosis, cerebral sinus thrombosis, stroke caused by systemic inflammation, hematological disorders (such as thalassemia, aplastic anemia), arterial dissection (such as carotid dissection), cardiovascular diseases (such as valvular heart disease, embolism due to atrial fibrillation)
▪ Location and size: Anterior or posterior circulation, cortical or subcortical
▪ Diagnostic neuroimaging: CT, CTA, MRI, MRA, DSA, etc.
▪ Cardiac examination: ECG, echocardiography, etc.
• **Pregnancy-/puerperium-associated variables**
▪ Week of gestation and trimester at stroke onset^a^
▪ Gestational hypertension
▪ Preeclampsia/eclampsia
▪ HELLP syndrome
▪ Gestational diabetes
▪ Frequency of previous pregnancy and pregnancy-/puerperium-associated stroke
• **Data on delivery**
▪ Type: Spontaneous vaginal delivery, cesarean section, or prenatal termination (induced abortion or labor)
▪ Week of gestation at delivery
• **Stroke management**
▪ Ischemic stroke
(1) Nonsurgical treatment (if with record dose and duration): anticoagulant (heparin, warfarin), antiplatelet (aspirin, clopidogrel), intravenous thrombolysis (alteplase, tenecteplase), etc.
(2) Surgical treatment: mechanical thrombectomy, etc.
▪ Hemorrhagic stroke
(1) Nonsurgical treatment: Supportive (blood pressure control, intracerebral pressure control), etc.
(2) Surgical treatment: Open surgery (clipping for IA, excision for AVM, hematoma evacuation), endovascular intervention (coiling for IA, embolization for AVM), ventriculopuncture drainage, etc.
▪ Time relationship with terminating the pregnancy (if with surgical treatment): after, before, or during the same period
• **Complications**
▪ Neurologic: Intracerebral rebleeding (hemorrhagic stroke), vasospasm or delayed cerebral ischemia, intracerebral hemorrhage (ischemic stroke), brain herniation, etc.
▪ Systemic: Chest infection, urinary tract infection, deep venous thrombosis, pulmonary embolism, gastrointestinal bleeding, myocardial ischemia, etc.
• **Offspring-related variables**
▪ Term at birth: Pre-term, full-term, or post-term
▪ Gender
▪ Birth date
▪ Birth weight
▪ Apgar scores at 1, 5, and 10 min
▪ Transfer to NICU (if with record etiology)

*BMI* body-mass index, *NIHSS* National Institute of Health stroke scale, *mRS* modified Rankin Scale, *RBC* red blood cell, *WBC* white blood cell, *PLT* platelet, *TC* total cholesterol, *HDL-C* high-density lipoprotein cholesterol, *TT* thrombin time, *PT* prothrombin time, *FIB* fibrinogen, *APTT* activated partial thromboplastin time, *SBP* systolic blood pressure, *DBP* diastolic blood pressure, *AVM* arteriovenous malformation, *IA* intracerebral aneurysm, *CT* computed tomography, *CTA* computed tomography angiography, *MRI* magnetic resonance imaging, *MRA* magnetic resonance angiography, *DSA* digital subtraction angiography, *ECG* electrocardiography, *NICU* neonatal intensive care unit

^a^The first trimester is defined as the period from conception to 12 + 0 weeks of gestation. The second trimester spans from 12 + 1 weeks to 28 + 0 weeks of gestation. The third trimester begins after 28 + 1 weeks of gestation. Puerperium is defined as the period within 6 weeks postpartum

### Follow-up schedule

All participants will be followed up through telephone interviews. The initial follow-up is scheduled to take place between December 2024 and February 2025. The follow-up phase will consist of three rounds, each lasting 3 months and conducted every 3 years. The specific follow-up schedule is as follows:

- (1) First follow-up: Conducted between December 2024 and February 2025, focusing on stroke recurrence, cardiovascular health indicators, and quality-of-life assessments. During this period, any newly occurring stroke events will be documented, including the nature and severity of recurrent strokes, and changes in cardiovascular risk factors, such as blood pressure and lipid levels, will be monitored.
- (2) Second follow-up: Conducted between December 2027 and February 2028, continuing with telephone interviews and standardized questionnaires to assess participants’ life quality, including emotional state and daily activity capacity, while monitoring stroke and cardiovascular health status
- (3) Third follow-up: Conducted between December 2030 and February 2031 to further observe late-stage stroke and cardiovascular disease, particularly focusing on the increased cardiovascular risk phase post-menopause.

If participants report a stroke recurrence during the follow-up period, researchers will obtain the hospitalization records through the following process to confirm the event: First, researchers will contact the participant’s attending physician or the relevant hospital to systematically request the hospitalization records and medical information. Second, relevant medical records will be reviewed to verify the reported recurrence event, including confirmation of diagnosis, treatment course, and length of hospitalization. Additionally, if necessary, researchers will request neuroimaging assessments (such as CT or MRI) to further confirm the nature and severity of the stroke.

### Study outcomes

This study will focus exclusively on the short-term follow-up outcomes for women and their offspring. Specifically, short-term outcomes are defined as events occurring during hospitalization, with particular attention to the following aspects:

1. Complications: Assessment of complications related to postpartum stroke, including the risk of stroke recurrence, cardiovascular complications (such as heart failure and arrhythmias), and infection risks (such as pulmonary or urinary tract infections)
2. Medical interventions: Documentation of treatments administered during hospitalization, including antiplatelet or anticoagulant therapies, antihypertensive medication, antibiotic use, and physical therapy interventions, to evaluate their effectiveness and patient response
3. Changes in health status: Close monitoring of overall health fluctuations during the hospital stay, including neurological function, blood pressure control, heart rate stability, and emotional state, to comprehensively assess health variations throughout treatment

#### Primary outcome

These are the following: For Aim 1, composite unfavorable outcome defined as *mRS* > 2 or EQ-5D index score < 0.7 at follow-up; for Aim 2, recurrent strokes confirmed by neuroimaging; and for Aim 3, the Apgar scores of newborns and results from Ages & Stages Questionnaires, Third Edition (ASQ-3) at follow-up.

#### Secondary outcome

These are the following: For Aim 1, unfavorable functional outcomes at discharge; for Aim 2, strokes occurring during subsequent pregnancies; and for Aim 3, the occurrence of intrauterine death, stillbirth, or critical illnesses requiring hospitalization in the offspring (see Table 3).

**Table 3:** Outcome measures^a^

• **Women**
***Short-term outcomes***
▪ Inhospital mortality
▪ Unfavorable functional outcome (assessed by mRS and EQ-5D) at discharge^b^
▪ Duration of hospitalization
***Long-term outcomes***
▪ **Recurrent stroke** (if with record frequency, interval, and etiology)
▪ **Unfavorable functional outcome at follow-up**^b^
▪ Mortality
▪ Stroke during subsequent pregnancy
• **Offspring**
***Short-term outcomes***
▪ **Neonatal asphyxia** (assessed by Apgar scores)^c^
▪ Intrauterine death or stillbirth
***Long-term outcomes***
▪ **Developmental status** (assessed by ASQ-3) **at follow-up**^d^
▪ Critical illness (if with record etiology)^e^

*mRS* modified Rankin Scale, *EQ-5D* EuroQol five dimensions questionnaire *ASQ-3* Ages & Stages Questionnaires, Third Edition

^a^Short-term outcomes are defined as events occurring during hospitalization, while long-term outcomes are defined as health status within 1-year post-discharge and during follow-up. All participants will be followed up through telephone interviews. The initial follow-up is scheduled to take place between December 2024 and February 2025. The follow-up phase will consist of three rounds, each lasting 3 months and conducted every 3 years

^b^Functional outcomes will be assessed using the modified Rankin Scale (mRS), with an mRS score greater than 2 defined as an adverse outcome

^c^Neonatal asphyxia will be assessed using Apgar scores at 1, 5, and 10 min, with scores of 8–10 defined as no asphyxia, 4–7 as mild asphyxia, and 0–3 as severe asphyxia

^d^The ASQ-3 categorizes developmental status into five subdomains: (1) Communication, (2) gross motor, (3) fine motor, (4) problem-solving, and (5) personal-social. Based on scores in each subdomain, results will be classified as “above,” “close to,” or “below” the threshold

^e^Critical illness is defined as the offspring requiring hospitalization due to a diagnosed disease, including neurological diseases, cardiovascular diseases, gastrointestinal diseases, respiratory diseases, urological diseases, hematological and immunological diseases, and other major disorders

Functional outcomes will be assessed using the modified Rankin Scale (mRS), with an mRS score greater than 2 defined as an adverse outcome. An independent assessment team, composed of professionals who did not participate in the patients’ treatment, will be responsible for evaluating functional outcomes during follow-up to ensure objectivity and reduce potential bias. Members of this assessment team will receive systematic training to ensure their proficiency in the assessment criteria and procedures of the mRS and EQ-5D questionnaires, thereby enhancing the reliability of the assessment results.

Specific assessment criteria include the following:

The assessor will conduct the mRS scoring for each patient during the follow-up period through standardized interviews and questionnaires combined with clinical observations. The assessor will use a unified mRS scoring guideline to ensure the consistency of scoring. The assessor will supervise and guide patients to take the test using the validated Chinese version of the EQ-5D5D questionnaire.

##### Assessment process

Assessors will document changes in patients’ functional status during follow-up, including self-reported and observed capabilities in activities of daily living, to accurately evaluate functional outcomes.

Neonatal asphyxia will be assessed using Apgar scores at 1, 5, and 10 min, with scores of 8–10 defined as no asphyxia, 4–7 as mild asphyxia, and 0–3 as severe asphyxia. The assessment will be conducted by healthcare personnel trained specifically in neonatal care to ensure the accuracy and consistency of the scores.

Developmental outcomes for offspring will be assessed using the Ages and Stages Questionnaire, Third Edition (ASQ-3), which is one of the most widely used tools for screening comprehensive developmental status in children aged 1 to 66 months. The ASQ-3 categorizes developmental status into five subdomains: (1) Communication, (2) gross motor, (3) fine motor, (4) problem-solving, and (5) personal-social. Based on scores in each subdomain, results will be classified as “above,” “close to,” or “below” the threshold. The assessment will be conducted by trained assessors to ensure the reliability and accuracy of the results [14].

Recurrent stroke and stroke during subsequent pregnancy are defined as events where women experience a recurrent stroke and a stroke during a subsequent pregnancy, respectively, both confirmed by corresponding medical or insurance records. Intrauterine death or stillbirth is defined as a dead fetus in the uterus at any trimester or the birth of a dead infant after 28 + 1 weeks of gestation. Critical illness is defined as the offspring requiring hospitalization due to a diagnosed disease, including neurological diseases (such as epilepsy and cerebral palsy), cardiovascular diseases (such as congenital heart disease), gastrointestinal diseases (such as necrotizing enterocolitis), respiratory diseases (such as allergic asthma), urological diseases (such as hypospadias), hematological and immunological diseases, and other major disorders.

### Sample size estimates

Formal power calculations were conducted using parameters from Chinese population studies. Based on the Health Study of Chinese Shanghai Women reporting a 1.45-fold stroke risk increase with high parity (≥ 5 pregnancies), we calculated a requirement of 268 ASPP cases and 804 matched controls (1:3 ratio) to detect similar associations (*α*= 0.05, 80% power) [15]. For longitudinal analysis of stroke recurrence, 456 ASPP cases are needed to identify a 40% risk reduction (90% power), anchored to China-specific stroke incidence data. Our target enrollment of 400 ASPP patients balances statistical rigor and feasibility, accounting for a 20% attrition rate, multicenter recruitment capacity (36 centers, ~ 11 cases/center/year), and subgroup analyses (e.g., cerebral venous thrombosis).

### Statistical analysis

Descriptive statistics will express continuous variables as means (±standard deviation) or medians (interquartile range) and categorical variables as proportions. Chi-square tests or Fisher’s exact tests, along with Student’s *t*-tests or Wilcoxon rank-sum tests, will be used to compare demographics and clinical characteristics between cases and controls. Chi-square tests will compare dichotomous outcomes, while *t*-tests or Mann-Whitney *U*-tests will compare quantitative outcomes. Kaplan-Meier curves and log-rank tests will evaluate survival analyses, with Cox proportional hazard models calculating hazard ratios and 95% confidence intervals (CIs). Multivariate logistic regression will identify independent predictors, and linear or generalized linear models will assess the impact of ASPP and other factors on delivery outcomes and the ASQ-3 results. First, logistic regression will calculate odds ratios and 95% CIs for recurrent stroke. Subgroup analysis will evaluate potential differences in endpoints for predefined subgroups (such as gestational hypertension, preeclampsia, HELLP syndrome, gestational diabetes, trimester, and type of delivery). Multiple imputation will address missing baseline data, with a *p*-value of < 0.05 considered statistically significant.

## Discussion

ASPP is a rare yet severe complication in pregnant and postpartum women, significantly impacting their prognosis and survival. Several studies have demonstrated the incidence rate, risk factors, and treatment trends associated with ASPP [16, 17]. However, due to the rarity of this condition, prior research has relied on national healthcare databases and the ICD-10-CM coding to extract variables and outcomes, which inevitably limits the collection of detailed information necessary for a comprehensive investigation. Therefore, data from a patient-level cohort is essential for addressing significant unresolved clinical issues related to ASPP.

The ASPP is a retrospective, observational, nationwide, multicenter research project aimed at exploring both the short-term and long-term outcomes of the offspring of ASPP patients and their future development. This study has several notable strengths. First, it benefits from access to detailed patient-level data, including baseline characteristics and outcomes for both the women and their offspring, such as stroke severity, neuroimaging characteristics, management strategies during hospitalization, and Apgar and ASQ-3 scores of the offspring. Second, this study does not rely on the ICD-10-CM coding and can thus avoid coding errors, such as misclassification and underreporting. Third, it is capable of collecting long-term recovery outcomes for ASPP patients, an achievement not previously attained in studies on this subject. Fourth, both Apgar and ASQ-3 scores will be collected to evaluate the short-term and long-term impacts of ASPP on offspring. The results in this regard will aid physicians in informing parents about the potential effects of ASPP on the future development of their children. Consequently, this study may provide evidence for the comprehensive management of ASPP and inform the long-term prognosis for both ASPP patients and their offspring.

It is particularly important to emphasize that the etiology of stroke in ASPP patients is diverse, with a significant impact from pregnancy-related hypercoagulability on stroke risk [18]. During pregnancy, hormonal fluctuations, especially increases in estrogen and progesterone, may lead to changes in coagulation mechanisms, thereby increasing the risk of thrombosis [19]. This physiological change may not only result in venous thrombosis during pregnancy but may also trigger arterial thrombosis and other cardiovascular events. Specifically, risk factors for pregnancy-related hypercoagulability include previous thrombotic events, obesity, advanced maternal age, and family history [20]. Hormonal changes during pregnancy may also affect the occurrence or rupture of intracranial aneurysms, thereby increasing the risk of stroke [17]. Preexisting cerebrovascular abnormalities, such as arteriovenous malformations, moyamoya disease, or untreated aneurysms, may further increase this risk [21]. These structural abnormalities usually require specialized management strategies, such as endovascular intervention versus conservative monitoring, which directly affect recurrence rates and long-term functional outcomes. This study included detailed neuroimaging reviews to document these potential diseases, enabling subgroup analyses of their impact on secondary prevention effectiveness and recurrence patterns during follow-up.

Other causes of stroke must also be considered, such as arterial dissection. This condition may be more common in young women, especially after childbirth, due to potential tears in the vascular intima [22]. Cardioembolic strokes can result from atrial fibrillation, valvular heart disease, or other heart issues. These conditions may be more frequent during pregnancy because of physiological changes and increased heart strain [23]. The complexity of these causes highlights the need for thorough risk assessment in pregnant women to identify and manage potential stroke risk factors.

In addition, managing ASPP requires individualized treatment plans based on the specific causes. When dealing with hypercoagulability, it is important to carefully consider the timing and choice of anticoagulant therapy to minimize risks to both the mother and fetus. Early neuroimaging assessments are also crucial in stroke management, as they help identify the location and nature of thrombi and guide treatment decisions.

The findings of this study should be interpreted within the context of potential limitations. Firstly, this is a retrospective study, which is subject to several inherent biases, such as recall and information bias, potentially jeopardizing its validity. Secondly, follow-up via telephone interviews may lead to a high dropout rate. Thirdly, concerns among ASPP patients regarding the privacy of their offspring’s information may result in a significant number being unwilling to cooperate, leading to selection bias. To address these issues, expanding the sample size by including more centers would be an effective solution for achieving statistical validity. Finally, although this study focuses on the Chinese population and mainly includes the Han ethnic group as the primary ethnic cohort, it employs two methodological measures, geographical heterogeneity and biological consistency, to ensure the generalizability of the research findings. The study included 22 provinces representing different socio-economic and medical resource conditions to ensure geographical heterogeneity. According to previous national studies, in the Chinese population, the key risk factors for ASPP, such as hypertension and coagulation dysfunction, show minimal differences across different ethnic groups, suggesting that the study subjects have biological consistency [24, 25].

## Conclusions

The ASPP study is a nationwide, multicenter, patient-level investigation that retrospectively encompasses ASPP patients and non-ASPP participants from 36 centers across China. This study aims to provide comprehensive information on the risk factors associated with ASPP, as well as its short-term and long-term prognoses and the risk of recurrent stroke, particularly during subsequent pregnancies. Furthermore, it will be the first study to explore whether this severe complication during pregnancy affects the future development of offspring.

## Data Availability

Not applicable.

## Abbreviations

ASPP: Acute stroke in pregnancy and puerperium
mRS: Modified Rankin Scale
EQ-5D: EuroQol five dimensions questionnaire
ASQ-3: Age & Stages Questionnaires, Third Edition
